# GURFAP: A Platform for Gene Function Analysis in *Glycyrrhiza Uralensis*

**DOI:** 10.3389/fgene.2022.823966

**Published:** 2022-04-12

**Authors:** Jiaotong Yang, Hengyu Yan, Yue Liu, Lingling Da, Qiaoqiao Xiao, Wenying Xu, Zhen Su

**Affiliations:** ^1^ Resource Institute for Chinese and Ethnic Materia Medica, Guizhou University of Traditional Chinese Medicine, Guiyang, China; ^2^ College of Agronomy, Qingdao Agricultural University, Qingdao, China; ^3^ College of Horticulture, Qingdao Agricultural University, Qingdao, China; ^4^ College of Life Sciences, Northwest Normal University, Lanzhou, China; ^5^ College of Biological Sciences, China Agricultural University, Beijing, China

**Keywords:** *Glycyrrhiza uralensis*, Platform, gene function analyses, co-expression network, glycyrrhizin

## Abstract

*Glycyrrhiza uralensis* (Licorice), which belongs to Leguminosae, is famous for the function of pharmacologic action and natural sweetener with its dried roots and rhizomes. In recent years, the whole-genome sequence of *G. uralensis* has been completed, which will help to lay the foundation for the study of gene function. Here, we integrated the available genomic and transcriptomic data of *G. uralensis* and constructed the *G. uralensis* gene co-expression network. We then annotated gene functions of *G. uralensis via* aligning with public databases. Furthermore, gene families of *G. uralensis* were predicted by tools including iTAK (Plant Transcription factor and Protein kinase Identifier and Classifier), HMMER (hidden Markov models), InParanoid, and PfamScan. Finally, we constructed a platform for gene function analysis in *G. uralensis* (GURFAP, www.gzybioinfoormatics.cn/GURFAP). For analyzed and predicted gene function, we introduced various tools including BLAST (Basic local alignment search tool), GSEA (Gene set enrichment analysis), Motif, Heatmap, and JBrowse. Our analysis based on this platform indicated that the biosynthesis of glycyrrhizin might be regulated by MYB and bHLH. We also took *CYP88D6*, *CYP72A154,* and *bAS* gene in the synthesis pathway of glycyrrhizin as examples to demonstrate the reliability and availability of our platform. Our platform GURFAP will provide convenience for researchers to mine the gene function of *G. uralensis* and thus discover more key genes involved in the biosynthetic pathway of active ingredients.

## Introduction

*Glycyrrhiza uralensis* (Leguminosae family), an herb medicine, is distributed all over the world, especially in the northwest and northeast of China (Yang et al., 2014). It is wildly used in traditional Chinese medicine with its dried roots and rhizomes for thousands of years (Tang et al., 2015). Ancient records in Shennong Materia Medica demonstrated that *G. uralensis* was good for relieving cough and reducing sputum, regulating painful menstruation and toxin resolving (Cheng et al., 2021). For example, compound licorice tablets (CPLTs) contain various ingredients including *G. uralensis* and have antitussive and expectorant effects (Hou and Sun, 2021). Modern pharmacological studies show that the active extract of *G. uralensis* has the function of anti-oxidant, anti-inflammatory, anti-tumor, anti-viral, liver protection, memory-enhancement, anti-aging, and so on (Kwon et al., 2020; Huan et al., 2021). Due to its antioxidant and anti-aging activities, *G. uralensis* is also widely used in cosmetics (Li et al., 2020; Yu et al., 2021). Some brands of cosmetics such as LANCOME, CHANEL, CLINIQUE, and Shiseido contain extracts of *G. uralensis*. In addition, *G. uralensis* is also wildly used in food processing as a natural non-caloric sweetener (DuBois and Prakash, 2012). With the research development, many chemical components of *G. uralensis* are gradually discovered, such as flavonoids, triterpenoid saponins, coumarin, and polysaccharides (Chengcheng Wang et al., 2020), which give it multiple pharmaceutical functions. Many drugs approved by China Food and Drug Administration (CFDA) contain the extract of *G. uralensis*, such as Licorice extract powder, Glycyrrhetinic acid and MgIG (Yang et al., 2017). Glycyrrhizin is one of the important components of *G. uralensis* to exert pharmacological effects and is used for the treatment of chronic hepatitis in Asian countries (Xu et al., 2016; Huan et al., 2021).

All the reasons above demonstrate that *G. uralensis* has good development opportunities and application prospects. However, increasing demand is easy to result in over-exploitation. Although the cultivated *G. uralensis* is wildly distributing in northwest China, the secondary metabolites had a lower level than that in wild samples (Wang et al., 2021). Therefore, exploring the molecular mechanism of secondary metabolites biosynthesis will improve the accumulation of secondary metabolites in cultivated *G. uralensis.*

In recent years, with the wide application of high-throughput sequencing technology in the field of life science, several medicinal plant genomes have been decoded, such as *Lonicera japonica* (Pu et al., 2020), *Gastrodia elata* (Yuan et al., 2018), *Catharanthus roseus* (Kellner et al., 2015), *Eriobotrya japonica* Lindl (Su et al., 2021), and *Carthamus tinctorius* (Wu et al., 2021), which provides an important guarantee for protection and development medicinal plants genomes.To better share genome information, genome functional databases of many medical plants are constantly published (She et al., 2019; Yang et al., 2020), which will be of great importance for gene functional research. She *et al* (She et al., 2019) collected genome sequence and 53 RNA-seq datasets of *Catharanthus roseus* from public databases and constructed the online croFGD database, then added miRNA-target pairs and several tools to predicted gene function. Yang *et al* (Yang et al., 2020) integrated genome, transcriptome and other relevant data of *Gastrodia elata* to annotate the gene function and identified gene families, and then constructed the online database GelFAP. At the same time, a variety of gene function analysis tools were introduced to GelFAP, which facilitated the research on gene function of *G. elata.* Xiao *et al* (Xiao et al., 2021) established the functional genome database (LjaFGD) of *Lonicera japonica*. In this platform, the researchers collected 77 transcriptome data to construct the co-expression network of *L. japonica*, and then they found that MYB and WRKY transcription factor family co-expressed with key enzyme genes in the biosynthesis of chlorogenic acid and luteolin. This database will provide ideas for studying the biosynthesis mechanism of chlorogenic acid and luteolin in *L. japonica.* In addition to the research about medicine plant databases, other plant databases had also been reported, such as Coriander Genomics Database (CGDB) (Song et al., 2020), Kiwifruit Genome Database (KGD) (Yue et al., 2020), Portal of Juglandaceae (PJU) (Guo et al., 2020), Malvaceae plants (MaGenDB) (Dehe Wang et al., 2020).

The whole-genome sequence of *G. uralensis* has been published (Mochida et al., 2017), which will provide more information for gene functional research. However, no comprehensive database of *G. uralensis* for gene functional mining and analysis is available for researchers. Therefore, combined with the genome and 35 transcriptome samples of *G. uralensis* from public platforms, we established an online gene functional platform, named GURFAP. In this platform, we constructed gene co-expression network and annotated gene function of *G. uralensis* by aligning with public platform. We also predicted the protein-protein interaction (PPI) network of *G. uralensis* based on the PPI network of *A. thaliana*. In addition, we introduced various analysis tools including basic local alignment search tool (BLAST), gene sets enrichment analysis (GSEA), motif analysis, and heatmap for researchers to explore gene functions. Based on GURFAP, we found that the biosynthesis of glycyrrhizin might be regulated by transcription factors such as MYB and bHLH, we also cited examples to demonstrate the reliability and availability of our platform. We hope this platform will provide convenience for the research about *G. uralensis* gene function.

## Materials and Methods

### Data Resource

Genome data was derived from *G. uralensis* genome database (http://ngs-data-archive.psc.riken.jp/Gur-genome/index.pl), including genome sequence, gene location file (gff3 file), transcript sequence, protein sequence. 35 transcriptome data samples of *G. uralensis* are from National Center for Biotechnology Information (NCBI, https://www.ncbi.nlm.nih.gov/) Sequence Read Archive (SRA, https://www.ncbi.nlm.nih.gov/sra/) and the detailed information is listed in Supplementary Table S1. Public protein sequences used for gene functional annotation are derived from non-redundant protein sequence database (NR, https://ftp.ncbi.nlm.nih.gov/blast/db/FASTA), cluster of orthologous groups of proteins (COG, https://www.ncbi.nlm.nih.gov/COG), the *Arabidopsis* information resource (TAIR, https://www.arabidopsis.org/) (Reiser et al., 2017), swissprot, and translated EMBL nucleotide sequence data library (TrEMBL) database (https://www.uniprot.org/downloads). Gene ontology (GO) functional annotation is based on AgriGOv2 (Tian et al., 2017). Kyoto Encyclopedia of Genes and Genomes (KEGG) (https://www.kegg.jp/) annotation is based on GhostKOALA (https://www.kegg.jp/ghostkoala/) (Kanehisa et al., 2016). Pfam domain annotation information comes from the Pfam database (http://pfam.xfam.org/) (El-Gebali et al., 2019).

### Gene Family Collection and Prediction

The Ethylene-responsive element binding factor-associated Amphiphilic Repression (EAR) motif-containing proteins were collected from PlantEAR (http://structuralbiology.cau.edu.cn/plantEAR) (Yang et al., 2018). Transcription factors (TF), transcription regulators (TR), and protein kinase (PK) family members were predicted by iTAK software (http://itak.feilab.net/cgi-bin/itak/index.cgi) (Yi et al., 2016). Ubiquitin proteases were predicted by HMMER software (Potter et al., 2018) based on the hidden Markov model files of conserved domains of ubiquitin proteases downloaded from ubiquitin and ubiquitin-like conjugation database (UUCD, http://uucd.biocuckoo.org/) (Gao et al., 2013). For cytochrome P450 (CYP450), we firstly collected all the CYP450 protein from public databases (http://drnelson.utmem.edu/CytochromeP450.html) (Nelson, 2009), and then predicted the homology between CYP450 protein and *G. uralensis* protein by InParanoid (bootstrap>60%) (Sonnhammer and Ostlund, 2015). Finally, we obtained the predicted CYP450 proteins of *G. uralensis.* To identify the carbohydrate-active enzymes (CAZy), we also collected the CAZy gene of *A.thinana* from the CAZy database (http://www.cazy.org/) (Lombard et al., 2014), and then matched the CAZy gene family to *G. uralensis* based on orthologous relationship.

### Transcriptome Data Processing

RNA-seq transcriptomic data samples of *G. uralensis* were obtained from NCBI SRA database. We used Hisat2 software (Kim et al., 2019) to map clean reads to the reference genome and then calculated the fragments per kilobase of exon model per million reads mapped (FPKM) values by the Stringtie software (Pertea et al., 2015). In addition, We used the RseQC package (Wang et al., 2012) and WigToBigwig (Lee et al., 2020) software to generate wiggle files for further display and analysis.

### Co-Expression network Construction

The gene expression matrix was constructed based on the FPKM expression values of each gene in each sample, and then the expression value of each gene was normalized by z-score method to calculate the Pearson Correlation Coefficient (PCC) between every two genes. Different PCC thresholds were set to construct co-expression networks. By evaluating the change of scale-free fit index and network density with the PCC value, the most appropriate PCC threshold was selected to construct the co-expression network, so that the constructed co-expression network was the most suitable for the scale-free network distribution with a relatively lower density.

### Protein-Protein Interaction network Construction

PPIs of *Arabidopsis* we previously collected from the BioGRID (http://thebiogrid.org/) (Oughtred et al., 2019), TAIR (Lamesch et al., 2012), and BAR (http://bar.utoronto.ca/welcome.htm) (Waese and Provart, 2017) were used to predict the PPI network of *G. uralensis*. The orthologous proteins between *Arabidopsis* and *G. uralensis* were predicted by InPranoid software (bootstrap>0.6), and then the PPI network of *Arabidopsis* was mapped to *G. uralensis* to construct its protein interaction network.

### Platform Construction and Visualization

We constructed the gene functional platform of *G. uralensis* based on classical LAMP (Linux, Apache, MySQL and PHP) architecture. Network was display by cytoscape.js (http://js.cytoscape.org/) (Franz et al., 2016) and heatmap was display by highcharts javascript (https://www.highcharts.com/).

### Gene Set Enrichment Analysis Tool

Gene set enrichment analysis (GSEA) is an analysis method for gene functional annotation of gene sets. PlantGSEA provides gene set enrichment analysis tool for various plants. Here, we used the same method to construct GSEA of *G. uralensis* and the background gene sets including GO annotation, KEGG annotation, and gene family.

### Motif Enrichment Analysis Tool

Cis-acting elements (Motif) enrichment analysis tool we used in the platform was as our previous research (Yang et al., 2020; Xiao et al., 2021). Motifs significantly enriched (*p*-value<0.05) were defined by calculating *p*-value and z-score as below formula:

$$Z=\frac{\bar{X}-\mu }{\sigma /\sqrt{n}}$$

$$p-\mathrm{value}=1-\mathrm{pnorm}\left(\bar{X},\mu ,\frac{\sigma }{\sqrt{n}}\right)$$

**‾X**) Number of matches to a motif in the promoter (3-kb) of candidate genes; **μ**) Average number of matches to the same motif in the promoter of 1000 random lists of genes; **σ)** Standard deviation of the motif from 1000 random selection numbers; **n)** Gene number. **Pnorm)** Distributed function of the normal distribution.

### Other Tools

In the platform, Online JBrowse (Buels et al., 2016) was used to display *G. uralensis* genes and transcriptome data. BLAST was used for sequence alignment. Heatmap analysis tool was used to display and compare the expression of genes in various transcriptome samples.

## Results

### Functional Annotation

**FIGURE 1:**
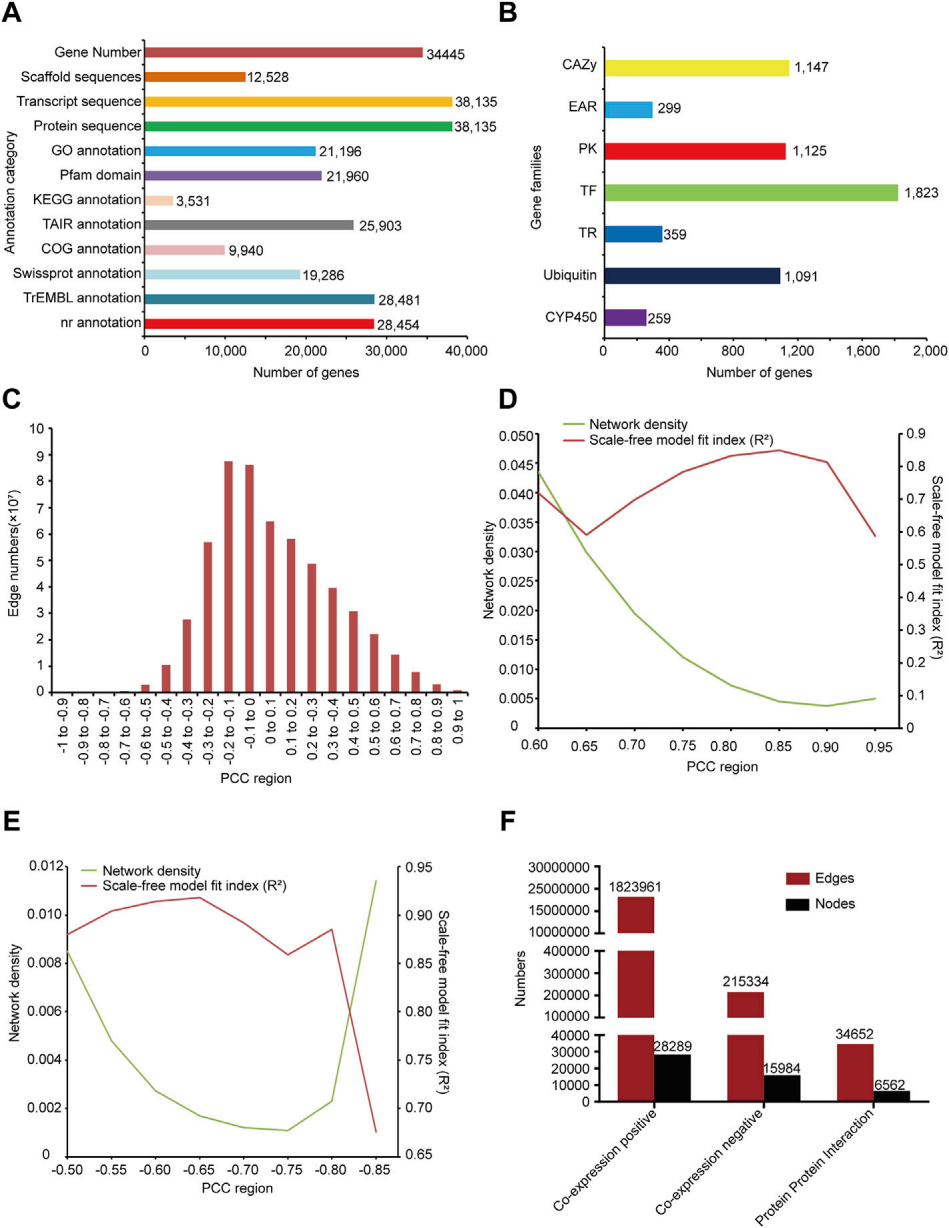
Overview of functional annotation and co-expression network constructed. **(A)** The number of sequences and annotated genes. **(B)** Classification of gene families and number of genes, including transcription factors (TFs), transcription regulators (TRs), protein kinases (PKs), EAR motif-containing proteins (EARs), cytochrome P450 (CYP450), Carbohydrate-Active enzymes (CAZys) and Ubiquitin proteases. **(C)** The diagram distributions of the gene-pairs as PCC changing. **(D)** Network density and scale-free model fit (R^2^) distribution in the positive co-expression network according to changing PCC. **(E)** Network density and scale-free model fit (R^2^) distribution in the negative co-expression network according to changing PCC. **(F)** Statistics of nodes and edges in the positive co-expression network, negative co-expression network, and PPI network.

We annotated 28454, 19286, 9940, 28481, 25903 genes respectively by comparison with protein bank of gene annotation database NR, Swissprot, COG, trEMBL, and TAIR (Reiser et al., 2017) (Figure 1A). KEGG annotations of 3531 genes were predicted by KEGG database (Figure 1A). 21196 gene GO term and annotation of *G. uralensis* were obtained from AgriGOv2 (Tian et al., 2017). Protein domains of 21960 genes were predicted by localized PfamScan software (Figure 1A).

### Gene Family Prediction and Collection

In the research, 1823 transcription factors, 359 transcriptional regulators, and 1125 protein kinases were predicted by iTAK software. UUCD database (Gao et al., 2013) provided the HMM profile of conserved domains of ubiquitin protease family, we used it to predict 1091 ubiquitin proteases of *G. uralensis.* Orthologous relationships can be used to predict gene families in different species, so we collected CYP450 and CAZy sequences from the cytochrome P450 homepage (Nelson, 2009) and CAZy database (Lombard et al., 2014) respectively, and predicted 259 CYP450 and 1147 CAZy genes of *G. uralensis via* InParanoid softward (Sonnhammer and Ostlund, 2015). In addition, 299 EAR motif-containing proteins were collected from PlantEAR (Figure 1B).

### Analysis Tools

We firstly introduced BLAST analysis tool in GURFAP. BLAST analysis can help users to analyze homology between specific sequences and transcripts or proteins of *G. uralensis.* We also provided gene set enrichment analysis tools (GSEA) (Yi et al., 2013) and motif enrichment analysis tools. For GSEA analysis, we provided GO, KEGG, and gene family as background gene sets. For motif analysis, we took the motif we collected as the analysis background for users to conduct motif enrichment analysis. In addition, we also built a genome browser for *G. uralensis* based on JBrowse (Buels et al., 2016) to demonstrate gene structure and transcriptome expression level. Heatmap analysis was added to visually show the expression values of candidate gene lists in different samples.

### Co-Expression Network

We first mapped reads from each RNA-seq sample to the genome of *G. uralensis* and screened the transcriptome with an overall mapping ratio more than 60% (Supplementary Table S1) and then calculated the expression level of each gene in all samples. The correlation between genes expression was calculated by the PCC algorithm to construct the gene co-expression network of *G. uralensis.* According to the PCC distribution result, we found that the correlation between most genes was not high, mainly concentrated in the middle part (Figure 1C), and with the increase or decrease of correlation, the number of gene pairs gradually decreased (Figure 1C). Biological networks are usually scale-free with low network density (Khanin and Wit, 2006; Broido and Clauset, 2019; Xiao et al., 2021). For the positive co-expression network, we evaluated and set the threshold of PCC in 0.6–0.95 to calculate the scale-free model fit index (R^2^) and network density respectively. We found that the scale-free fit index was the highest with PCC>0.85 and was the lowest with PCC < −0.65 (Figures 1D,E). Therefore, we chose PCC>0.85 and PCC < −0.65 to construct the positive and negative co-expression networks of *G. uralensis* (Figures 1D,E)*.* Finally, we obtained the positive co-expression network including 28289 nodes and 1823961 edges, and the negative co-expression network including 15984 nodes and 215,334 edges (Figure 1F).

### PPI network

We predicted the orthologous relationship of *Arabidopsis* and *G. uralensis* proteins using the Inparanoid software (bootstroop>0.6) (Sonnhammer and Ostlund, 2015), and then map the PPI network of *Arabidopsis* to *G. uralensis* according to their orthologous relationship. Finally, we obtained PPI network of *G. uralensis* with 6562 nodes and 34652 edges (Figure 1F).

### Expression View of Co-expression network

To make full use of the collected transcriptome data, we conducted a comparative analysis of transcriptome data and obtained differential expression genes (Supplementary Figure S1). SRP053019 is a set of data containing four RNA-seq samples, moderate drought stress, and control (repeat twice). Comparative analysis showed that 2803 genes were significantly up-regulated and 2595 genes were significantly down-regulated under moderate drought stress (Supplementary Figure S1A). We also conducted comparative analysis of SRP065514 (Supplementary Figure S1B), DRP000996 (Supplementary Figure S1C), SRP215420 (Supplementary Figure S1D) and SRP188776 (Supplementary Figure S1E). Combining with differential expression genes and network display, we constructed the expression view of co-expression network. Based on this expression view, users can obtain up-regulation and down-regulation genes in gene networks under specific conditions.

### Platform Framework

**FIGURE 2:**
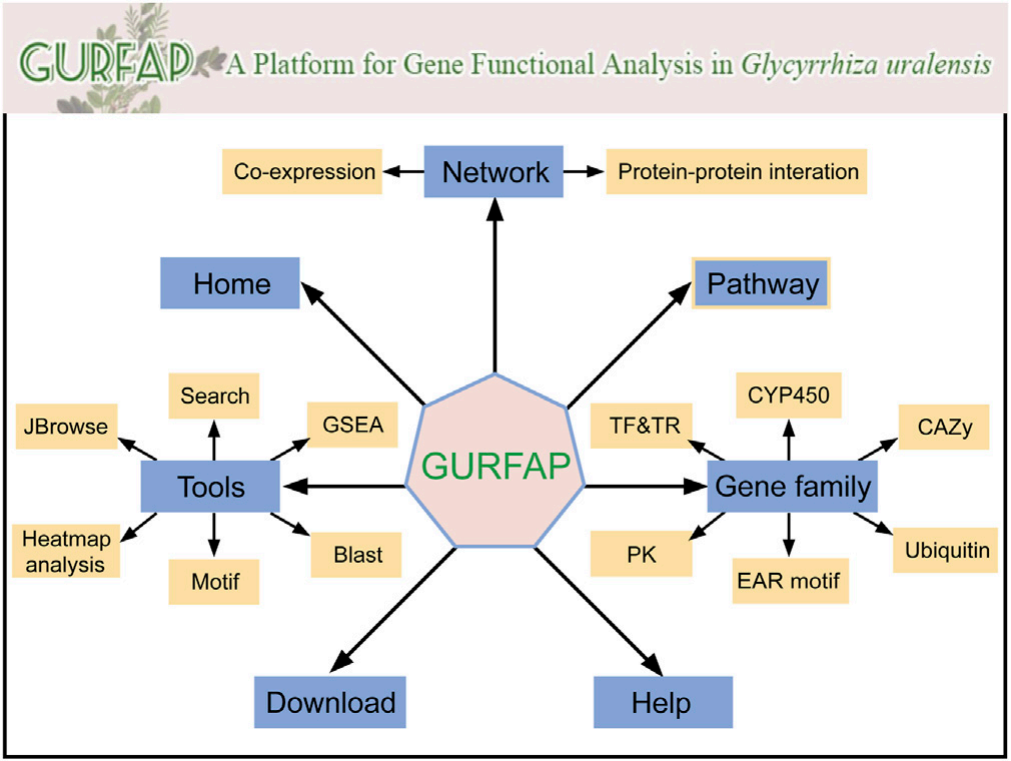
Organizational chart for GURFAP. Network section provides co-expression network and PPI network. The gene family section contains 6 different gene family classifications. The tools section also contains 6 analysis tools. Home contains the introduction of *G. uralensis* and the platform. Pathway, Download and Help become a separate part.

Integrating relevant annotation information and analysis tools, we constructed a platform for gene functional analysis in *G. uralensis.* In the platform, seven sections are displayed with different functions (Figure 2). The home section mainly contains the introduction of *G. uralensis* and the platform. The network section contains the search of co-expression network and PPI network. The tools section contains search, GSEA, BLAST, Motif, Heatmap Analysis, and JBrowse. The pathway section contains genes of pathway annotated by KEGG. Gene family section contains information about transcription factors (TF), transcription regulators (TR), protein kinase (PK), EAR motif-containing proteins (EAR), cytochrome P450 (CYP450), Carbohydrate-Active enzymes (CAZy), and Ubiquitin proteases families. Download and Help section are individual part that provides users with download information and operation information.

### Function Application

#### Co-Expression Network Analysis of Key Enzyme Genes in Glycyrrhizin Biosynthesis

**FIGURE 3:**
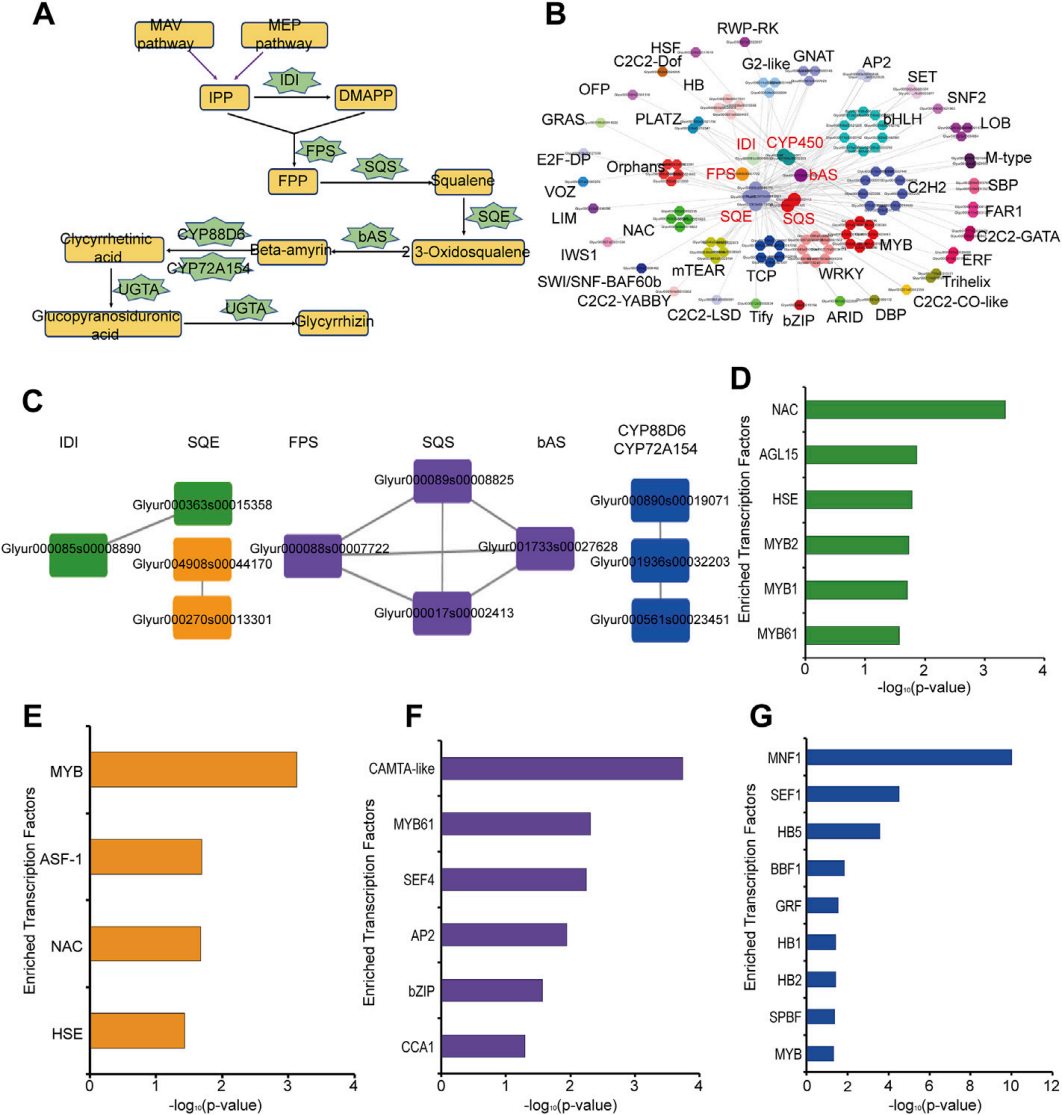
Analysis of key enzymes in glycyrrhizin biosynthesis pathway in *Glycyrrhiza* uralensis Fisch. **(A)** glycyrrhizin biosynthesis pathway and key enzymes. **(B)** Co-expression relationship between transcription factors (TFs) and key enzymes in glycyrrhizin biosynthesis. **(C)** The analysis results of the co-expression relationship between key enzymes, which can be divided into 4 groups, the first subgroup is the co-expression relationship between 1 IDI and 1 SQE (green, #389339), the second subgroup represents co-expression relationship between 2 SQE (orange, #f3951b), the third subgroup represents the co-expression relationship of 1FPS, 2 SQS and 1bAS (purple, #674b9c), the fourth subgroup represents co-expression relationship in 3 CYP450 genes (blue, #204fa2). **(D)** Motif enrichment analysis results of the first group (green, #389339). **(E)** Motif enrichment analysis results of the second group (orange, #f3951b). **(F)** Motif enrichment analysis results of the third group (purple, #674b9c). **(G)** Motif enrichment analysis results of the fourth group (blue, #204fa2).

Glycyrrhizin is one of the main pharmacological components in *G. uralensis* and its biosynthetic pathways have been relatively clear (Xu et al., 2016). Based on the KEGG signaling pathway annotation, we constructed the glycyrrhizin synthesis pathway, including the key enzymes isopentenyl diphosphate isomerase (IDI), farnesyl pyrophosphate synthase (FPS), squalene synthase (SQS), squalene monooxygenase or epoxidase (SQE), bamyrin synthetase (bAS), cytochrome P450 monooxygenases (CYP450), and glucuronosyltransferase (UGAT) (Figure 3A, Supplementary Table S2). Here we obtained 1 IDI, 1 FPS, 5 SQE, 3 SQS, and 1 bAS based on KEGG annotation. In addition, Seki *et al* found that two CYP450 were involved in glycyrrhizic acid synthesis (Seki et al., 2008; Seki et al., 2011). Xu *et al* found that one UGTA was involved in the biosynthesis of glycyrrhizin (Xu et al., 2016). According to the co-expression network analysis of these key enzymes, we found that these key enzymes are co-expressed with many transcription factors, such as MYB, WKRY, bHLH, and ERF (Figure 3B). Therefore, these transcription factors may play an important role in the synthesis of glycyrrhizin in *G. uralensis.* In addition, we found co-expression relationships among these key enzymes, which were divided into four different subgroups (Figure 3C). The first subgroup is the co-expression relationship between 1 IDI and 1 SQE (green, #389339), the second subgroup represents the co-expression relationship between 2 SQE (orange, #f3951b), the third subgroup represents 1FPS, 2 SQS and 1bAS are co-expressed (purple, #674b9c), the fourth subgroup represent the co-expressed relationship in 3 CYP450 genes (blue, #204fa2) (Figure 3C). To explore the upstream regulatory factors of these key enzyme genes with co-expression relationships, we separately analyzed the four groups genes by motif enrichment analysis tool as described in the materials and methods section, and found that many transcription factors were enriched in their promoter regions. MYB, NAC, HSE, and AGL15 are enriched in the first groups (Figure 3D), MYB, NAC, and HSE are also enriched in second group (Figure 3E), bZIP, MYB, CCA1 are enriched in third group (Figure 3F) and MYB, HB etc., are existed in the last group (Figure 3G). This suggests that these transcription factors may play a regulatory role in the synthesis of glycyrrhizin.

#### Functional Analysis of Key Enzyme Genes in Glycyrrhizin Biosynthesis

**FIGURE 4:**
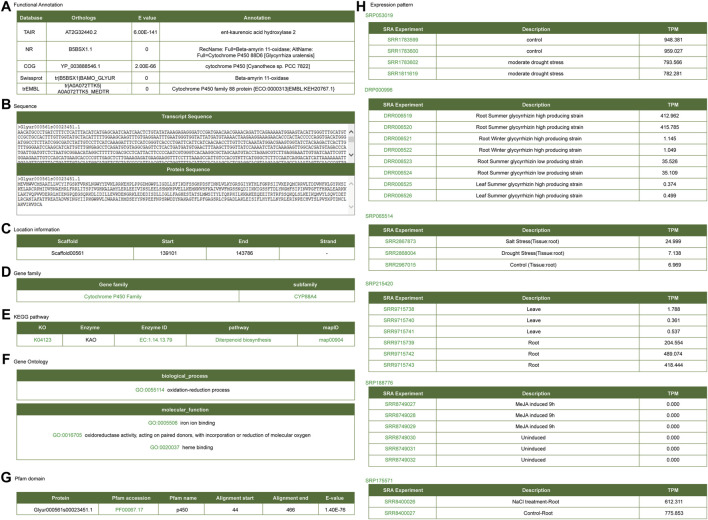
Gene detail interface of *CYP88D6* gene. **(A)** Functional annotation. **(B)** Transcript and protein sequence. **(C)** Gene location information. **(D)** Gene family. **(E)** KEGG signal pathway. **(F)** GO annotation. **(G)** Protein domain. **(H)** Expression level in different RNA-seq samples.

*CYP88D6*, *CYP72A154*, and *bAS* are key enzymes involved in the biosynthesis of glycyrrhizin (Hayashi et al., 1999; Seki et al., 2008; Shibuya et al., 2009; Seki et al., 2011). Here, we took these key enzymes as examples to introduce the application of the platform. Functional annotations for *CYP88D6* exhibited on the gene detail page are including annotated information of public platform (Figure 4A), gene sequence information (Figure 4B), gene structure information (Figure 4C), gene family (Figure 4D), KEGG metabolic pathway information (Figure 4E), GO annotation information (Figure 4F), protein domain information (Figure 4G), gene expression profile information (Figure 4H). This gene was annotated as ent-kaurenoic acid hydroxylase or Cytochrome P450 family 88 protein. Ent-kaurenoic acid hydroxylase is a key enzyme in the synthesis pathway of terpenoids and glycyrrhizin is one of the triterpenoid saponins. Therefore, annotation analysis indicated that *CYP88D6* may be involved in the synthesis process of glycyrrhizin. The structure information section provided the link of JBrowse, from which one can link to the JBrowse interface to check the gene structure and expression.

**FIGURE 5:**
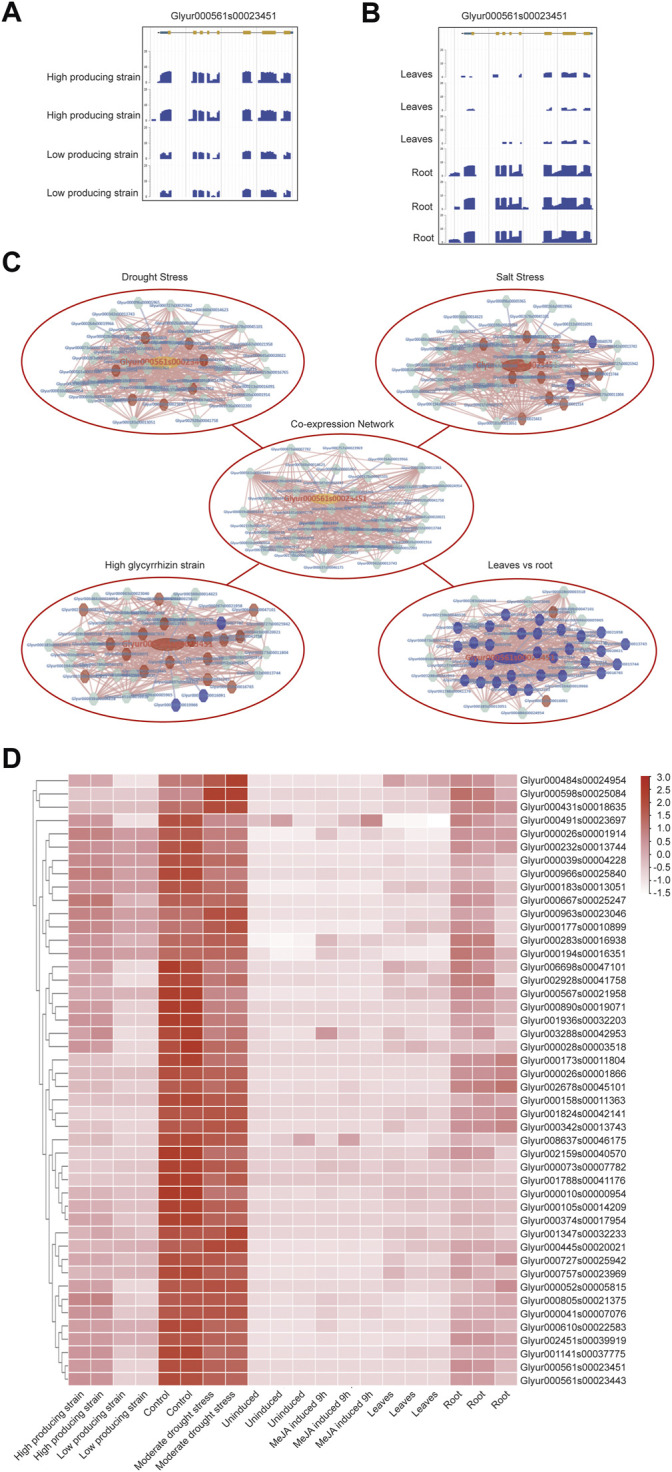
Functional analysis for *CYP88D6* gene. **(A)** CYP88D6 expression in high producing and high producing lines of *glycyrrhizin uralensis* in summer displayed by JBrowse. **(B)** CYP88D6 expression in leaves and root of *glycyrrhizin uralensis* displayed by JBrowse. **(C)** The up-regulation and down-regulation of CYP88D6 co-expressed genes in different conditions, red represent up-regulation and blue represent down-regulation. **(D)** The expression levels of CYP88D6 co-expressed genes in different samples displayed by heatmap.

KEGG annotation information indicated that *CYP88D6* might be involved in the triterpenoid synthesis. Related studies have shown that the accumulation of glycyrrhizin will be up-regulated under drought stress (Xie et al., 2018), and the accumulation of glycyrrhizin is in the roots (Li et al., 2011). Next, we conducted further analysis of the *CYP88D6* gene. The expression of this gene in different transcriptomes was analyzed and it was found that the expression of this gene in the lines with high glycyrrhizin yield was higher than that in the lines with low glycyrrhizin yield (Figure 5A). It was also found that the expression of this gene in roots was significantly higher than that in leaves (Figure 5B). Those results are consistent with the accumulation trend of glycyrrhizin. According to the co-expression network of *CYP88D6,* we found that this gene had a positive co-expression relationship with 46 genes and a negative co-expression relationship with 4 genes (Figure 4C). GSEA analysis of co-expressed genes shows that genesets related to Ent-kaurenoic acid hydroxylase, starch catabolic process significantly enriched (Supplementary Figure S2). Several genes in the *CYP88D6* co-expression network were significantly up-regulated under drought stress, and also significantly up-regulated in the root and glycyrrhizin high producing lines (Figure 4C). The results are consistent with the accumulation trend of glycyrrhiza. Finally, we analyzed the heatmaps of *CYP88D6* expression in different samples and found the same results with network analysis (Figure 4D). Therefore, our analysis results indicated that *CYP88D6* might be involved in the biosynthesis of glycyrrhizin in roots.

We searched for *CYP72A154* and *bAS* genes in the platform respectively and obtained annotated information including gene function, gene sequence, gene structure information, gene family, KEGG metabolic pathway information, GO annotation information, protein domain information, and gene expression profile information (Supplementary Figures S3, S4). JBrowse was used to analyze the expression profile and found that *CYP72A154* and *bAS* genes were expressed higher in high-yield lines than that in low-yield lines (Supplementary Figures S5A, S6A), and they were also expressed higher in roots than that in leaves (Supplementary Figures S5B, S6B). These results were consistent with the accumulation trend of glycyrrhizin (Li et al., 2011). In addition, we used JBrowse to analyze the expression of *CYP72A154* under drought stress and salt stress, and found no significant change in *CYP72A154* expression compared with the control (Supplementary Figures S5C,D). We also analyzed the co-expression networks of *CYP72A154* and *bAS* under different conditions, and found that the co-expression genes were significantly up-regulated in the high yield lines of glycyrrhizin, and significantly down-regulated in the leaves (Supplemenatry Figure S6C and Supplementary Figure S5E). However, the expression differences of *CYP72A154* under drought and salt stress are not obvious (Supplementary Figure S5E).

## Discussion

*G. uralensis* is a traditional Chinese medicinal herb, its flavonoids, triterpenoid saponins, coumarin, and polysaccharides are important functional components (Chengcheng Wang et al., 2020). The release of the genomic data for *G. uralensis* provides us a considerable gene resource to study underlying biosynthesis mechanisms of bioactive components. However, there is no comprehensive platform for functional genetic analysis and mining in *G. uralensis*. To ease use by researchers, we developed a publicly available platform called GURFAP, and many tools have been introduced to analyze gene functions. We hope that researchers in the field can use the platform to achieve the new discovery of their study.

Our platform currently offers a searchable system for users, allowing a quick function search and browse of any genes. The obtained information includes structural annotations, functional annotations, gene expression profiles, and co-expression networks of genes, which can provide references for biologists to study the function of *G. uralensis* genes. All useful tools are publicly available, such as JBrowse (Buels et al., 2016), heatmap analysis, and GSEA, and can also contribute to the functional analysis of genes. JBrowse can be used to display omics data. By integrating the JBrowse tool, users can view and analyze gene expression more intuitively. The platform provides the Heatmap tool to display genes in the gene co-expression network or user-defined gene sets. The GSEA tool can also analyze the gene set provided by the user. In addition to analyzing the genes that have a co-expression relationship with candidate genes, it can also be used to analyze user-defined gene sets, such as differentially expressed genes, which are helpful for researchers to carry out the mining of gene function. There is a certain gap between the information provided by the platform and other databases, and the platform data information and analysis tools still need to be continuously upgraded.

Gene co-expression networks of our platform are based on the correlation of gene expression. Co-expression networks in numerous plants has been developed to infer the gene co-functional relationships, such as studies concerning the co-expression network in *Paeonia lactiflora* have mining genes related to Oleate Desaturase and Photosynthesis (Sheng et al., 2020). Genes in a co-expression network have similar expression patterns, so we reasoned that they may be regulated by the same transcription factors. Our previous co-expression network and motif analysis discovered the biosynthesis pathways of chlorogenic acid and luteolin in *L. japonica* might be regulated by MYB, bHLH, WRKY (Xiao et al., 2021). Here, gene co-expression network and motif analysis show transcription factors might participate in the regulation of glycyrrhizin biosynthesis. Glycyrrhizin is a triterpene saponin, whereas MYB, bHLH, ERF, WKRY involved in the regulation of the biosynthesis of triterpenoids have already been reported (Yao et al., 2020). This analysis method can provide a reference for users to study other secondary metabolite synthesis pathways.

On the other hand, three cases were used to present the usage of our platform. Comprehensive analysis of *CYP88D6*, *CYP72A154*, and *bAS* suggests that it might involve in regulation of glycyrrhizic acid biosysthesis, which had also been reported (Seki et al., 2008). This indicates that our platform is of certain usability and reliability. Although we used *CYP88D6*, *CYP72A154*, and *bAS* to illustrate the usability and reliability of this platform, our data came from public platforms, and most of the information was obtained by computational methods, such as functional annotation information and GO annotation information, etc. Therefore, the platform information is only for reference. To explore the possible functions of genes, molecular biology experiments are still needed.

We believe that omics data of *G. uralensis* will keep increasing with the development of high-throughput sequencing technologies and the continuous reduction in their cost. Timely and effective collection and the process will assist researchers in their project. Our future work will prioritize effective integration of omics information, and update platform timely. Interesting users can obtain the URL at www.gzybioinformatics.cn/GURFAP.

## Data Availability

The original contributions presented in the study are included in the article/Supplementary Material, further inquiries can be directed to the corresponding authors.
