# Supplementary material for: GURFAP: A Platform for Gene Function Analysis in Glycyrrhiza Uralensis
Source: Front Genet. 2022 Apr 12;13:823966. doi: 10.3389/fgene.2022.823966 (PMC9039005; doi:10.3389/fgene.2022.823966)
Supplement: Supplementary file 1 [file DataSheet1.docx]

***Supplementary Material***

**GURFAP: a platform for gene function analysis in *Glycyrrhiza uralensis***

Jiaotong Yang^1^, Hengyu Yan^3^, Yue Liu^4^, Lingling Da^5^, Qiaoqiao Xiao^1*^, Wenying Xu^2*^, Zhen Su^2*^

^1^Resource Institute for Chinese and Ethnic Materia Medica, Guizhou University of Traditional Chinese Medicine, Guizhou 550025, China

^2^State Key Laboratory of Plant Physiology and Biochemistry, College of Biological Sciences, China Agricultural University, Beijing 100193, China

^3^College of Agronomy, Qingdao Agricultural University, Qingdao 266109, China.

^4^College of Horticulture, Qingdao Agricultural University, Qingdao 266109, China.

^5^College of Life Sciences, Northwest normal university, Lanzhou 730070, China.

***Correspondence:**

**Qiaoqiao Xiao** (xqqiao2021@163.com)

**Wenying Xu** ( x_wenying@yahoo.com)

**Zhen Su**(zhensu@cau.edu.cn)

**Supplementary Tables**

**Table S1**. Summary of RNA-seq datasets collected.

| Sample | Description | Mapping rate |
| --- | --- | --- |
| DRR006519 | Root Summer glycyrrhizin high producing strain | 93.04 |
| DRR006520 | Root Summer glycyrrhizin high producing strain | 92.87 |
| DRR006521 | Root Winter glycyrrhizin high producing strain | 90.99 |
| DRR006522 | Root Winter glycyrrhizin high producing strain | 90.74 |
| DRR006523 | Root Summer glycyrrhizin low producing strain | 89.84 |
| DRR006524 | Root Summer glycyrrhizin low producing strain | 89.64 |
| DRR006525 | Leaf Summer glycyrrhizin high producing strain | 92.24 |
| DRR006526 | Leaf Summer glycyrrhizin high producing strain | 92 |
| DRR066062 | Unclear | 93.22 |
| SRR1783599 | Control | 89.89 |
| SRR1783600 | Control | 89.88 |
| SRR1783602 | moderate drought stress | 88.47 |
| SRR1811619 | moderate drought stress | 88.4 |
| SRR2537378 | Unclear | 87.92 |
| SRR2867873 | Salt Stress(Tissue:root) | 88.26 |
| SRR2868004 | Drought Stress(Tissue:root) | 88.46 |
| SRR2967015 | Control (Tissue:root) | 88.84 |
| SRR8400026 | Gu-NaCl | 90.31 |
| SRR8400027 | Gu-CK | 88,69 |
| SRR8468083 | Unclear | 89.64 |
| SRR8749027 | MeJA induced 9h | 91.23 |
| SRR8749028 | MeJA induced 9h | 91 |
| SRR8749029 | MeJA induced 9h | 90.26 |
| SRR8749030 | Uninduced | 88.74 |
| SRR8749031 | Uninduced | 89.25 |
| SRR8749032 | Uninduced | 90,05 |
| SRR9202035 | H0-1 | 90.05 |
| SRR9202036 | C3-4 | 88.01 |
| SRR9202037 | H1 | 90.01 |
| SRR9715738 | Leave | 88.94 |
| SRR9715739 | Root | 88.34 |
| SRR9715740 | Leave | 90.46 |
| SRR9715741 | Leave | 91.03 |
| SRR9715742 | Root | 88.37 |
| SRR9715743 | Root | 86.08 |

**Table S2**. Putative key enzyme genes involved in glycyrrhizin biosynthesis.

| Gene ID | Enzyme Name |
| --- | --- |
| Glyur000085s00008890 | IDI |
| Glyur000088s00007722 | FPS |
| Glyur000771s00029724 | SQE |
| Glyur004908s00044170 | SQE |
| Glyur002907s00033935 | SQE |
| Glyur000363s00015358 | SQE |
| Glyur000270s00013301 | SQE |
| Glyur000017s00002413 | SQS |
| Glyur000089s00008825 | SQS |
| Glyur001733s00027628 | bAS |
| Glyur000561s00023451 | CYP88D6 |
| Glyur001936s00032203 | CYP72A154 |
| Glyur000740s00028701 | UGTA |

**Supplementary Figures**


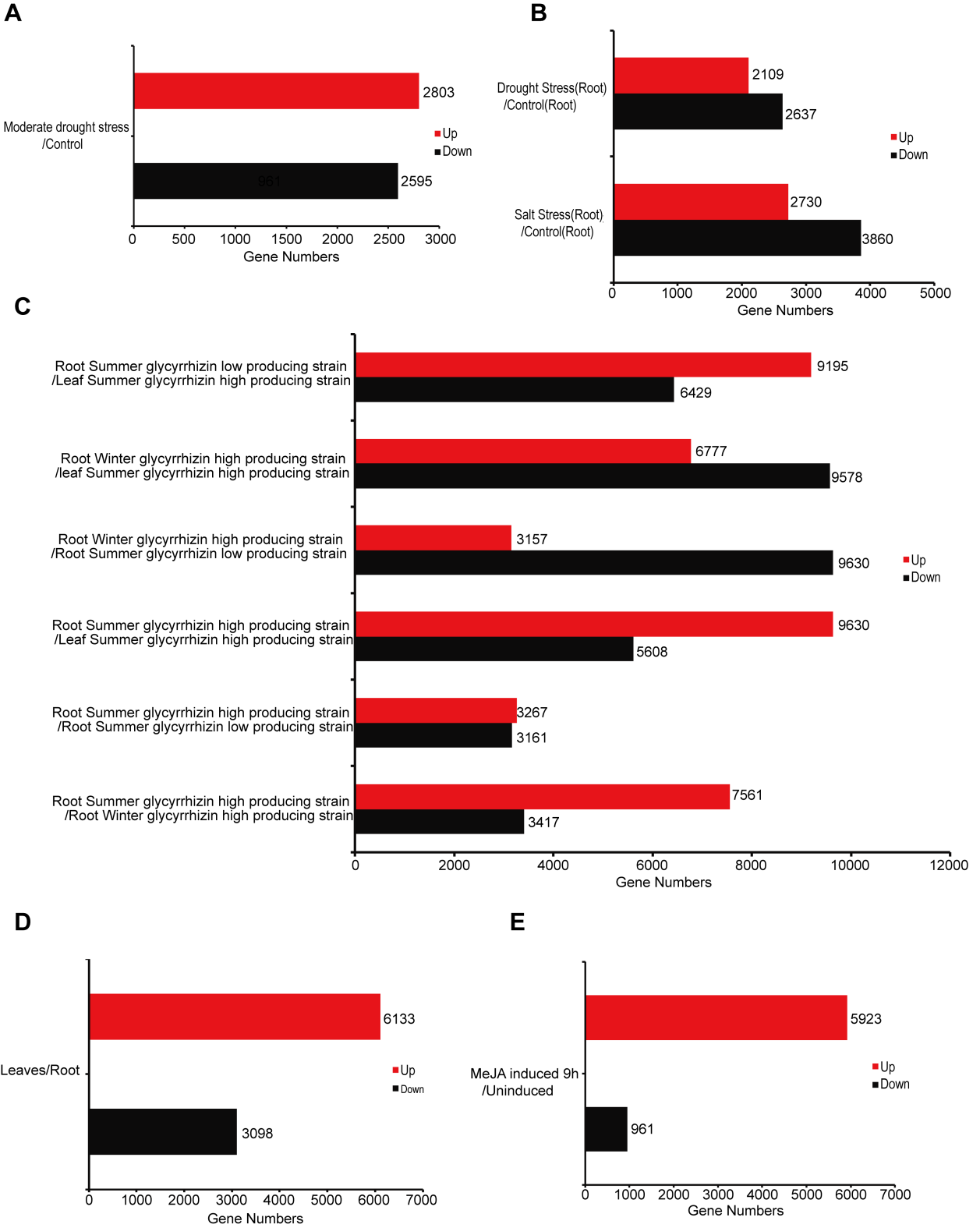


**Figure S1.** Number of significant differential expressed genes in different samples. (A) Moderate drought stress vs control (SRA accession number: SRP053019) (B) Salt Stress vs control, drought stress vs control in root (SRA accession number: SRP065514). (C) Comparison of RNA-sample in different glycyrrhizin producing strain (SRA accession number: DRR006519-DRR006526). (D) Leaves vs Root (SRA accession number: SRP215420). （F）MeJA induced 9h vs uninduced (SRA accession number: SRP188776).


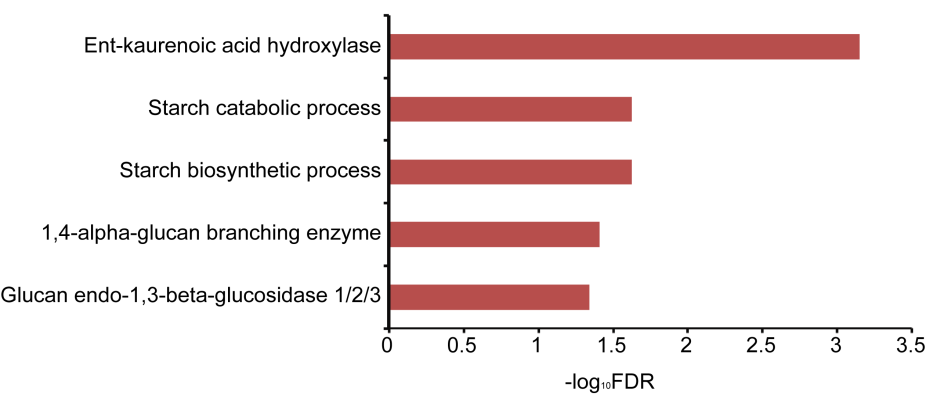


**Figure S2.** The results of genesets enrichment analysis on co-expressed genes of *CYP88D6*.


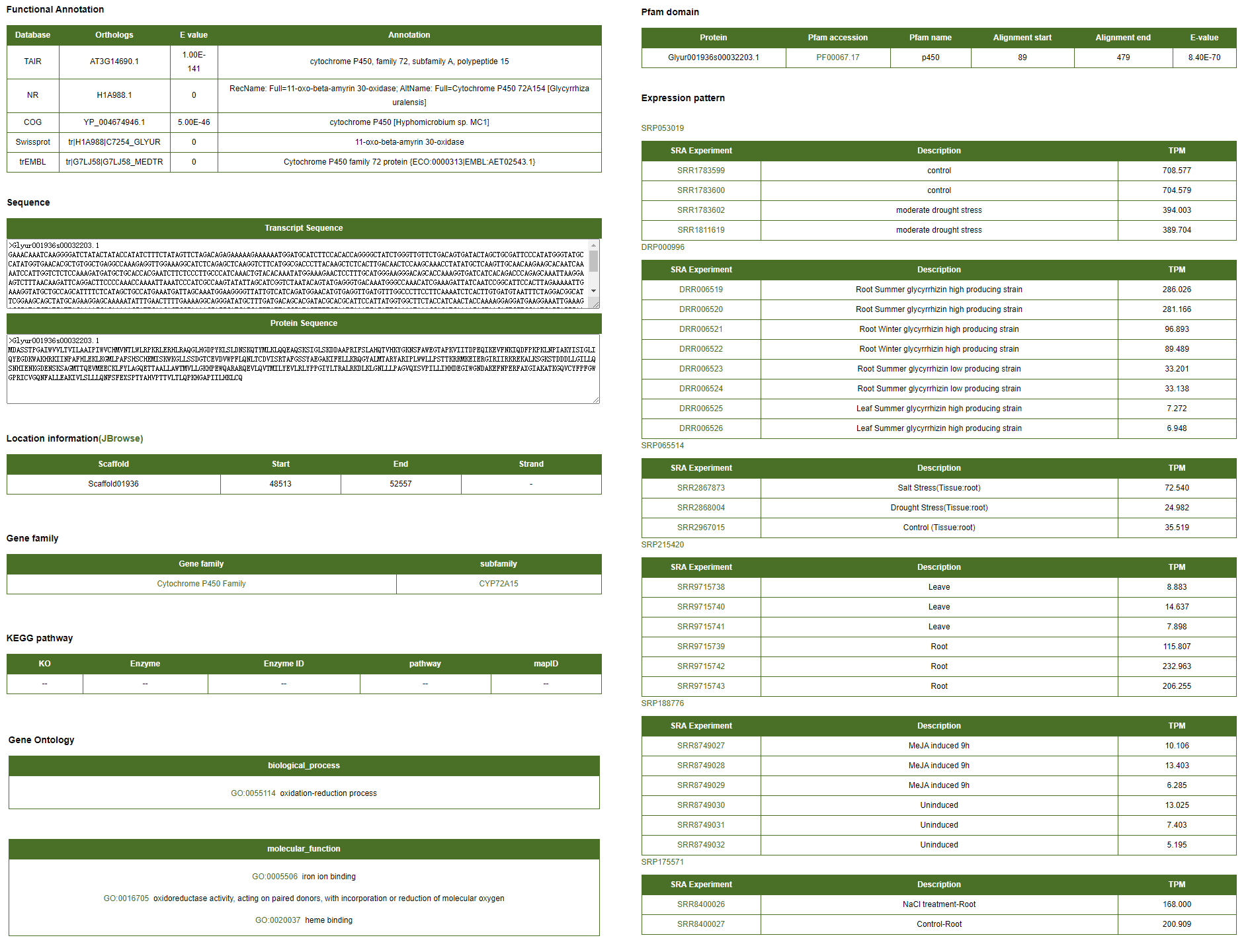


**Figure S3.** Gene detail interface of *CYP72A154*, including functional annotation, transcript and protein sequence, gene location information, gene family, KEGG signal pathway, GO annotation, protein domain and expression level in different RNA-seq samples.


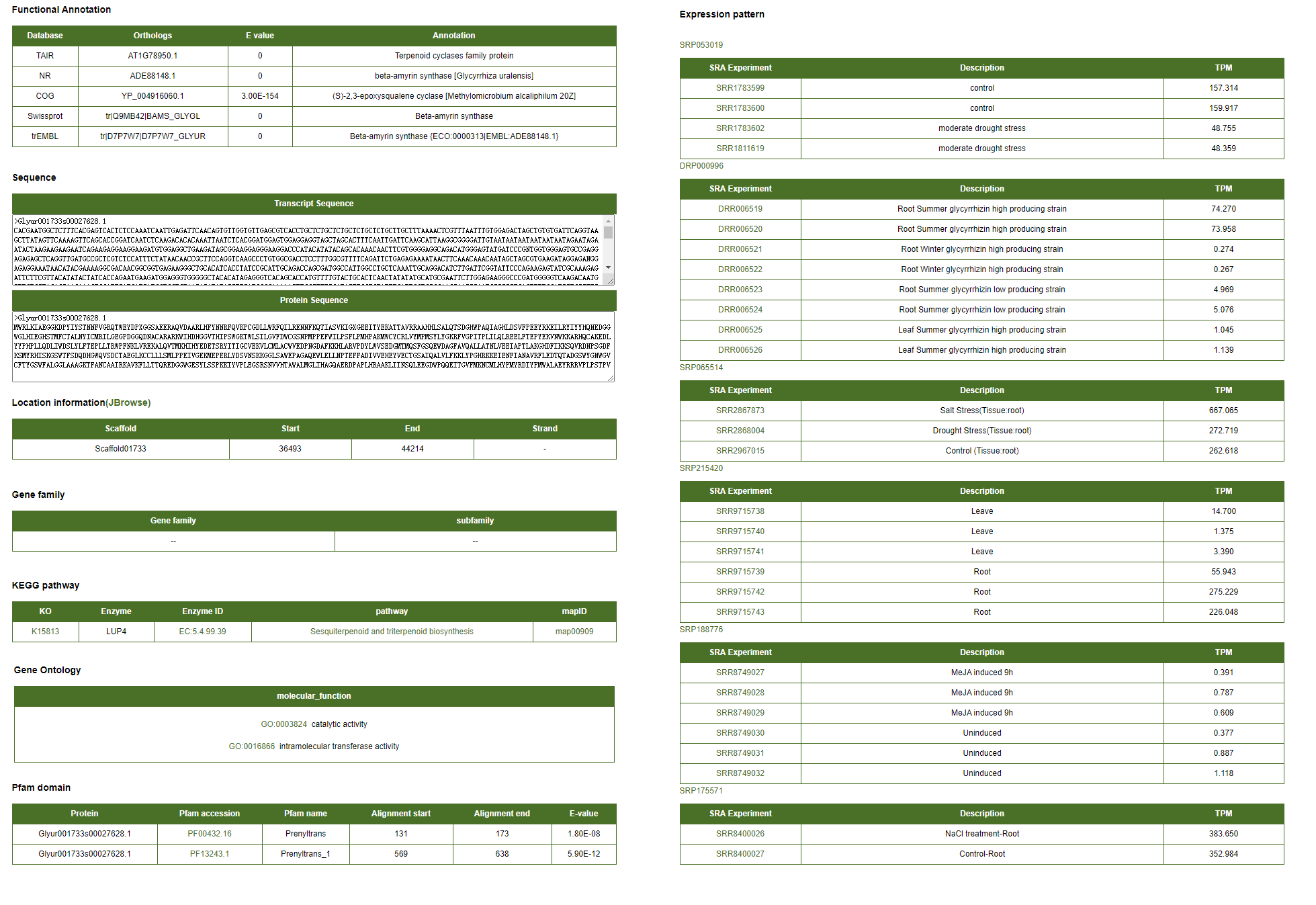


**Figure S4.** Gene detail interface of *bAS*, including functional annotation, transcript and protein sequence, gene location information, gene family, KEGG signal pathway, GO annotation, protein domain and expression level in different RNA-seq samples.


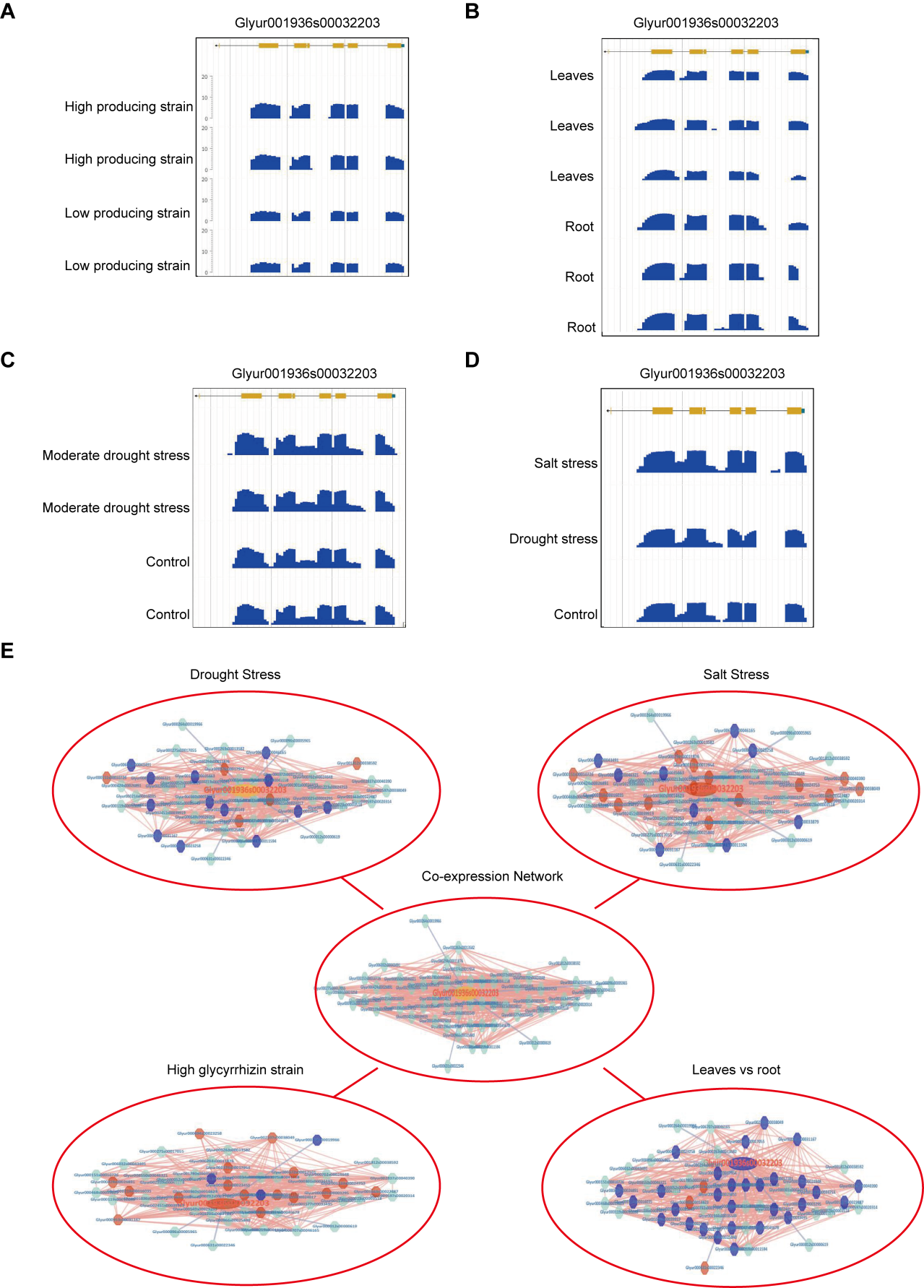


**Figure S5.** Functional analysis for *CYP72A154* gene. (A) *CYP72A154* expression in high producing and low producing lines of glycyrrhizin uralensis in summer displayed by JBrowse. (B) *CYP72A154* expression in leaves and root displayed by JBrowse. (C) *CYP72A154* expression in moderate drought stress and control condition displayed by JBrowse. (D) *CYP72A154* expression in salt, drought and control displayed by JBrowse. (E) The up-regulation and down-regulation of *CYP72A154* co-expressed genes in different conditions, red represent significant up-regulation and blue represent down-regulation.


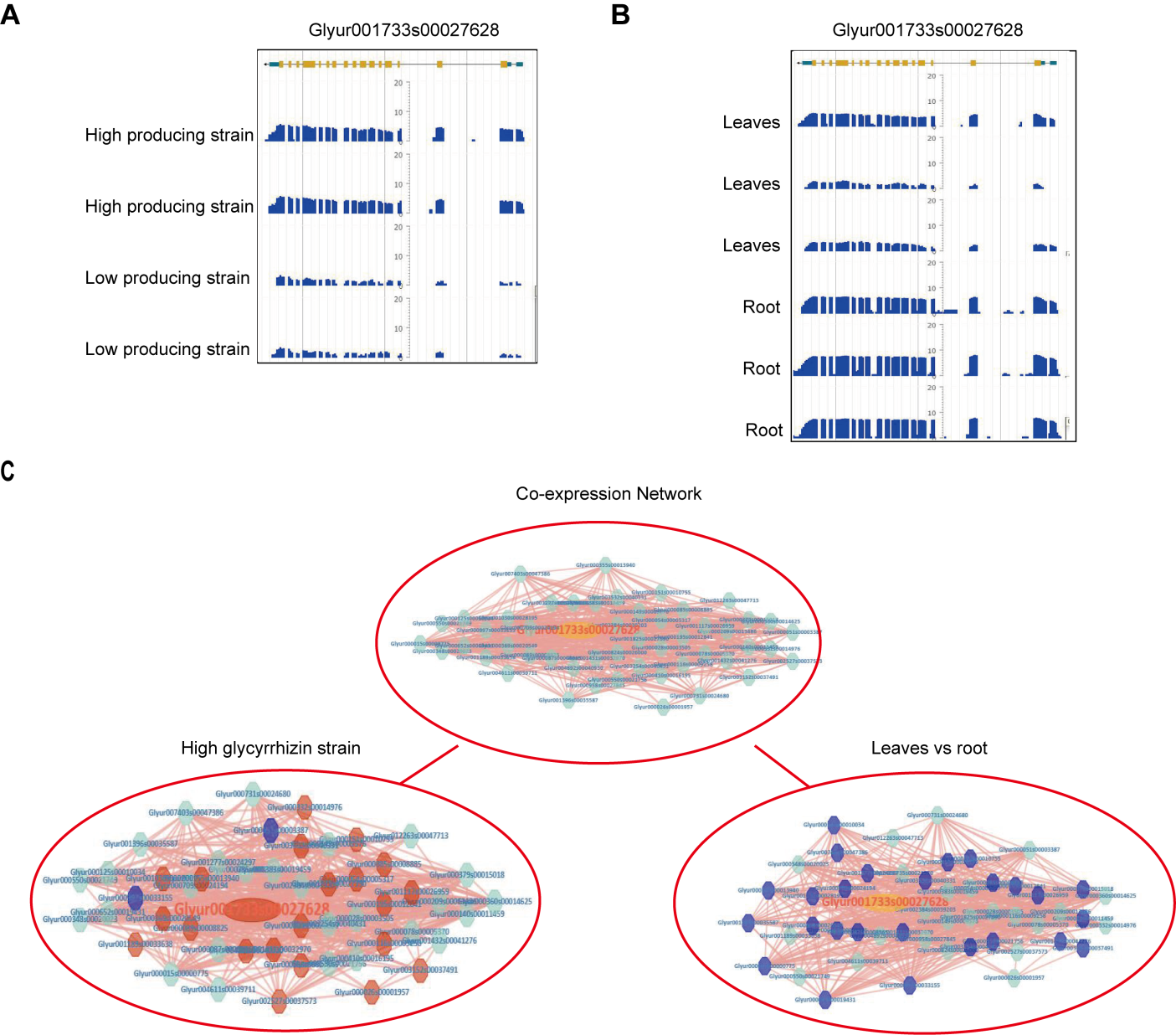


**Figure S6.** Functional analysis for *bAS* gene. (A) *bAS* expression in high producing and low producing lines of glycyrrhizin uralensis in summer displayed by JBrowse. (B) *bAS* expression in leaves and root displayed by JBrowse. (C) The up-regulation and down-regulation of *bAS* co-expressed genes in high glycyrrhizin strain and leaves, red represent significant up-regulation and blue represent down-regulation.
